# Systemic sarcoidosis complicated of acute renal failure: about 12 cases

**DOI:** 10.11604/pamj.2015.22.75.6237

**Published:** 2015-09-30

**Authors:** Madiha Mahfoudhi, Habiba Mamlouk, Sami Turki, Adel Kheder

**Affiliations:** 1 Internal Medicine A Department, Charles Nicolle Hospital, Tunis, Tunisia

**Keywords:** Sarcoidosis, acute renal failure, interstitial nephritis, granuloma

## Abstract

The sarcoidosis is a systemic granulomatosis affecting most frequently the lungs and the mediastinum. An acute renal failure reveals exceptionally this disease. It's a retrospective study implicating 12 cases of sarcoidosis complicated of acute renal failure. The aim of this study is to determine epidemiological, clinical, biological and histological profile in these cases and then to indicate the interest to consider the diagnosis of sarcoidosis in cases of unexplained renal failure. Extra-renal complications, therapeutic modalities and the outcome were determined in all patients. Our series involved 12 women with an average age of 40 years. Biological investigations showed an abnormal normocalcemia in 7 cases, a hypercalcemia in 5 cases, a hypercalciuria in 10 cases and polyclonal hypergammaglobulinemia in 7 cases. An acute renal failure was found in all patients with a median creatinin of 520 umol/L. For all patients, the renal echography was normaln however, the kidney biopsy showed tubulo-interstitial nephritis. The extra-renal signs highlighting pulmonary interstitial syndrome in 5 cases, a sicca syndrome in 4 cases, mediastinal lymph nodes in 2 cases, a lymphocytic alveolitis in 3 cases, an anterior granulomatous uveitis in 2 cases and a polyarthritis in 5 cases. Five patients benefited of hemodialysis. The treatment consisted of corticosteroid in all cases. The follow up was marked by complete resolution of clinical and biological signs. The diagnosis of renal sarcoidosis must be done quickly to prevent renal failure.

## Introduction

Sarcoidosis is a systemic granulomatous disease which preferentially involves the lung and mediastinum. A revealing acute renal failure is rare and a cause of positive diagnosis lateness [1]. It is usually due to abnormal calcium balance or parenchymal involvement. Renal biopsy allows the diagnosis by finding specific impairment. The prognostic factor for renal survival in sarcoidosis is the early diagnosis and response to treatment [1–3].

## Methods

This is a retrospective study which collected 12 cases of sarcoidosis complicated by acute renal failure hospitalized in Internal medicine A department of Charles Nicolle Hospital. The study consisted of identifying epidemiological data (age, gender), clinical symptoms (renal and extra-renal signs), biological signs (serum calcium level, urinary calcium, proteinuria / 24h, serum creatinine level) and histological results (renal biopsy), which confirmed the diagnosis. A study of the therapeutic and progressive profile (clinical and biological signs) was performed in all cases. The purpose of this study was to determine the epidemiological, clinical, biological and histological characteristics in 12 patients with acute renal failure complicating systemic sarcoidosis, and eventually to emphasize the interest to consider sarcoidosis for unexplained renal failure cases.

## Results

This study collected 12 cases with an average age was 40 years, ranging from 19 years to 71 years. Clinical examination found a preserved general state, apyrexia and normal blood pressure in all patients. No cases of peripheral lymphadenopathy or cutaneous sarcoid were mentioned. Cardiac, respiratory and neurological examinations were normal. Tuberculin skin test was negative in 8 cases. Laboratory tests showed an acute renal failure with a median creatinine 520 umol / L and creatinine values ranging from 138 to 1490 mmol / L, an inflammatory syndrome in 12 cases, an abnormal normocalcemia in 7 cases, a hypercalcemia in 5 cases with an average of 2.8 mmol /L, a hypercalciuria in 10 cases, a polyclonal hypergammaglobulinemia in 7 cases and a negative proteinuria in 8 cases. Proteinuria was low in 4 patients with an average of 0.3 g / 24h. Hypokalemia and metabolic acidosis were mentioned in 6 patients. The angiotensin-converting enzyme was elevated in 8 cases. Renal ultrasound was normal in all patients. Renal biopsy performed in all cases showed tubulo-interstitial nephritis in all patients, with the presence of giant cells in 2 cases and associated with giant cell granuloma in 6 cases. Extra-renal signs were: pulmonary interstitial syndrome in 5 cases, sicca syndrome in 4 cases, mediastinal lymphadenopathy infracentimétriques in 2 cases, lymphocytic alveolitis in 3 cases, anterior granulomatous uveitis in 2 cases and polyarthritis in 5 cases. According to the clinical, biological and histological results, the diagnosis of sarcoidosis complicated of acute renal failure has been retained. Five patients benefited of hemodialysis to severe renal impairment. All patients received steroids, at a dose of 1 mg/kg/day with a gradual degression, since they had active inflammatory kidney damage. The outcome was marked by improvement of serum creatinine levels and then normalization of renal function in all patients and serum calcium level in pathological cases. No new recurrence of renal sarcoidosis was observed in all our series with mean recoil of 5 years.

## Discussion

Sarcoidosis is a granulomatous giant cell non-caseating and multisystemic disease of unknown etiology. Mediastinal and pulmonary localizations is the most common [1]. Renal involvement in sarcoidosis is rare but can be severe by progressing to irreducible and end stage of renal failure. It is most often the result of disorders of calcium metabolism inducing calcium renal deposits [2]. The parenchymal involvement is frequently tubulointerstitial nephritis. Glomerular lesions remain exceptional including essentially membranous glomerulonephritis, rarely amyloidosis and exceptionally IgA nephropathy [1, 3]. Renal failure associated to hypokalemia and metabolic acidosis can be the biological signs of tubulo- interstitial nephropathy as in the case of 6 patients in our series. Renal biopsy has a very significant interest to the diagnosis of sarcoidosis especially in the absence of other extra-renal signs. At histology, the lesions associated interstitial cellular infiltrates and tubular inflammation. The presence of non-caseating granulomas, which is not constant, is highly suggestive of sarcoidosis diagnosis [1, 4]. In the series of Löffler U et al, including 27 cases, non-granulomatous tubulo-interstitial nephritis was the most common histological entity (44%), followed by granulomatous interstitial nephritis (30%), IgA glomerulonephritis (26%) and nephrocalcinosis (11%) [1]. Granulomatous tubulo-interstitial nephritis in sarcoidosis manifests rarely as acute renal failure feature [5, 6]. In fact, these lesions are often asymptomatic, and the prevalence is higher in autopsy series [1, 5]. Some rare cases of isolated sarcoidosis interstitial nephritis without visceral involvement had been published [1, 7, 8]. Granulomatous tubulo-interstitial nephritis secondary to sarcoidosis is a formal indication for systemic therapy because it threatens the renal functional outcome [1, 5, 9]. Corticosteroids remain the first line treatment of kidney damage. The dose and duration is variable and not codified. Recovery is more rapid and complete if the treatment was initiated at the early inflammatory stage and prior to fibrosis process [1, 9, 10]. In case of cortico-resistance, an immunosuppressor should be administrated. Corticosteroids usually give a rapid improvement of hypercalcemia, renal function, and complete resolution of extrarenal signs is the case of our patients. The prognosis of sarcoidosis is usually good with spontaneous remission but the chronic aspect can be seen in one third of cases and may be associated then with one or more organ dysfunctions [1, 7, 9, 10]. A chronic kidney failure may be secondary to sarcoidosis especially in cases of diagnostic and therapeutic delay lateness.

## Conclusion

Renal involvement in sarcoidosis is uncommon but severe and can lead to chronic kidney failure. Early diagnosis and adapted treatment allows preserving renal function.
